# Attenuation of chemokine receptor function and surface expression as an immunomodulatory strategy employed by human cytomegalovirus is linked to vGPCR US28

**DOI:** 10.1186/s12964-016-0154-x

**Published:** 2016-12-12

**Authors:** Theresa Frank, Anna Reichel, Olav Larsen, Anne-Charlotte Stilp, Mette M. Rosenkilde, Thomas Stamminger, Takeaki Ozawa, Nuska Tschammer

**Affiliations:** 1 Department of Chemistry and Pharmacy, Emil Fischer Center, University of Erlangen-Nuremberg, Erlangen, Germany; 2 Institute for Clinical and Molecular Virology, University of Erlangen-Nuremberg, Erlangen, Germany; 3 Department of Neuroscience and Pharmacology, Laboratory for Molecular Pharmacology, Faculty of Health and Medical Sciences, University of Copenhagen, Copenhagen, Denmark; 4 Department of Chemistry, School of Science, The University of Tokyo, Tokyo, Japan; 5 Present Address: NanoTemper Technologies GmbH, Floessergasse 4, 81069 Munich, Germany

**Keywords:** Viral G protein-coupled receptor US28, Chemokine receptor CXCR4, Constitutive activity, Bioluminescence resonance energy transfer, Bioluminescence complementation, Signaling crosstalk

## Abstract

**Background:**

Some herpesviruses like human cytomegalovirus (HCMV) encode viral G protein-coupled receptors that cause reprogramming of cell signaling to facilitate dissemination of the virus, prevent immune surveillance and establish life-long latency. Human GPCRs are known to function in complex signaling networks involving direct physical interactions as well as indirect crosstalk of orthogonal signaling networks. The human chemokine receptor CXCR4 is expressed on hematopoietic stem cells, leukocytes, endothelial and epithelial cells, which are infected by HCMV or display reservoirs of latency.

**Results:**

We investigated the potential heteromerization of US28 with CXCR4 as well as the influence of US28 on CXCR4 signaling. Using Bioluminescence Resonance Energy Transfer and luciferase-complementation based methods we show that US28 expression exhibits negative effects on CXCR4 signaling and constitutive surface expression in HEK293T cells. Furthermore, we demonstrate that this effect is not mediated by receptor heteromerization but via signaling crosstalk. Additionally, we show that in HCMV, strain TB40E, infected HUVEC the surface expression of CXCR4 is strongly downregulated, whereas in TB40E-delUS28 infected cells, CXCR4 surface expression is not altered in particular at late time points of infection.

**Conclusions:**

We show that the vGPCR US28 is leading to severely disturbed signaling and surface expression of the chemokine receptor CXCR4 thereby representing an effective mechanism used by vGPCRs to reprogram host cell signaling. In contrast to other studies, we demonstrate that these effects are not mediated via heteromerization.

**Electronic supplementary material:**

The online version of this article (doi:10.1186/s12964-016-0154-x) contains supplementary material, which is available to authorized users.

## Plain English Summary

Some herpesviruses like human cytomegalovirus encode viral G protein-coupled receptors. These membrane receptors facilitate dissemination of the virus and often prevent immune surveillance. As we demonstrate in this work, the G protein-coupled receptor US28 of HCMV is able to severely disturb the signaling and surface expression of the human chemokine receptor CXCR4 and thus limit the immune response.

## Background

Certain herpesviruses like human cytomegalovirus (HCMV), Epstein-Barr virus (EBV) and Kaposi’s sarcoma-associated herpesvirus (KSHV) are known to encode viral G protein-coupled receptors (vGPCRs) [1, 2]. These vGPCRs had most probably been hijacked from the human genome as they resemble human GPCRs in structure and function. HCMV-encoded vGPCRs have previously been shown to interact with the signaling machinery of the host cell in a remarkably efficient manner [3]. This reprogramming of cell signaling by vGPCRs is often aimed at facilitating dissemination of the virus, preventing immune surveillance and establishing life-long latency [4]. HCMV encodes four vGPCRs, US27, US28, UL33 and UL78, among which US28 is the best-characterized. US28 plays a crucial role in the viral life cycle by promoting viral spread [5] and by activating the immediate early HCMV promoter [6], which is necessary for the transactivation of other viral genes. US28, which is constitutively active, can also bind to a wide range of chemokines [7], possibly acting as a “chemokine sink” to reduce immune responses at the site of inflammation [8]. Alternatively, the constitutive or chemokine-induced signaling activities of US28 may modulate intracellular signaling pathways consequently promoting virus replication. In addition, US28 was reported to act as a HIV coreceptor in certain cell types [9] and has been associated with pathogenic processes leading to atherosclerosis [10].

Host responses to viral infections involve complex interactions between chemokines and other cytokines that provide key communication signals resulting in the effective development of innate and adaptive immunity. Thus, innate immune responses are critical in limiting viral spread and averting virus-induced disease. The human chemokine receptor CXCR4 is a promising target for manipulation by vGPCRs as it is expressed on cells, which are infected by HCMV or display reservoirs of latency [11]. CXCR4 is specific for *stromal cell*-*derived factor*-*1α* (SDF-1α or CXCL12) and is highly expressed on hematopoietic stem and progenitor cells (HSPCs) in the bone marrow niche as well as on differentiated circulating blood cells [11]. It serves as a coreceptor for the cell entry of HIV [12], highly contributes to trafficking and homeostasis of human immune cells, stem cell homing in tissue regeneration [13], but also tumorigenesis and progression of various types of cancer [14–16]. CXCR4 is prone to function in various homo- and heteromeric complexes to deploy its differential effects as revealed by various crystal structures and additional methods [17–19]. Importantly, CXCR4 has been associated with vGPCR-mediated manipulation of the chemokine receptor homeostasis. The Epstein-Barr virus - encoded vGPCR BILF1 was found to attenuate CXCL12-induced CXCR4 signaling by scavenging Gα_i_-proteins and impairing CXCL12 binding to CXCR4. Interestingly, the G protein-coupling deficient mutant BILF1-K^3.50^A affected CXCL12 - induced signaling less effectively, indicating that BILF1 - mediated CXCR4 inhibition is a consequence of its constitutive activity [20]. Additionally, it was reported that the HCMV - encoded vGPCRs UL33 and UL78 modulate CXCR4 signaling, surface expression as well as its HIV coreceptor activity [3]. In these reports, the observed manipulations of CXCR4 signaling and surface expression were mainly attributed to a direct physical contact or heteromerization of CXCR4 with the viral GPCRs BILF1, UL33 and UL78.

As GPCRs can physically affect each other’s signaling by forming heteromeric complexes [21], we thoroughly investigated the possibility of physical interaction or heteromerization of the vGPCR US28 with the human chemokine receptor CXCR4. Indeed, US28 seems to employ a subtler but nevertheless very effective way to influence CXCR4 signaling. Our data support the assumption that the observed attenuation of the CXCR4 surface expression and signaling in the presence of US28 is partly attributed to the high constitutive activity of US28. We believe that the G protein-dependent constitutive signaling of US28 leads to indirect signaling crosstalk via shared intracellular signaling networks, which results in disturbed chemokine receptor signaling and reduced surface expression.

## Results

### US28 abates chemokine-induced G protein-mediated signaling of CXCR4

**Fig. 1:**
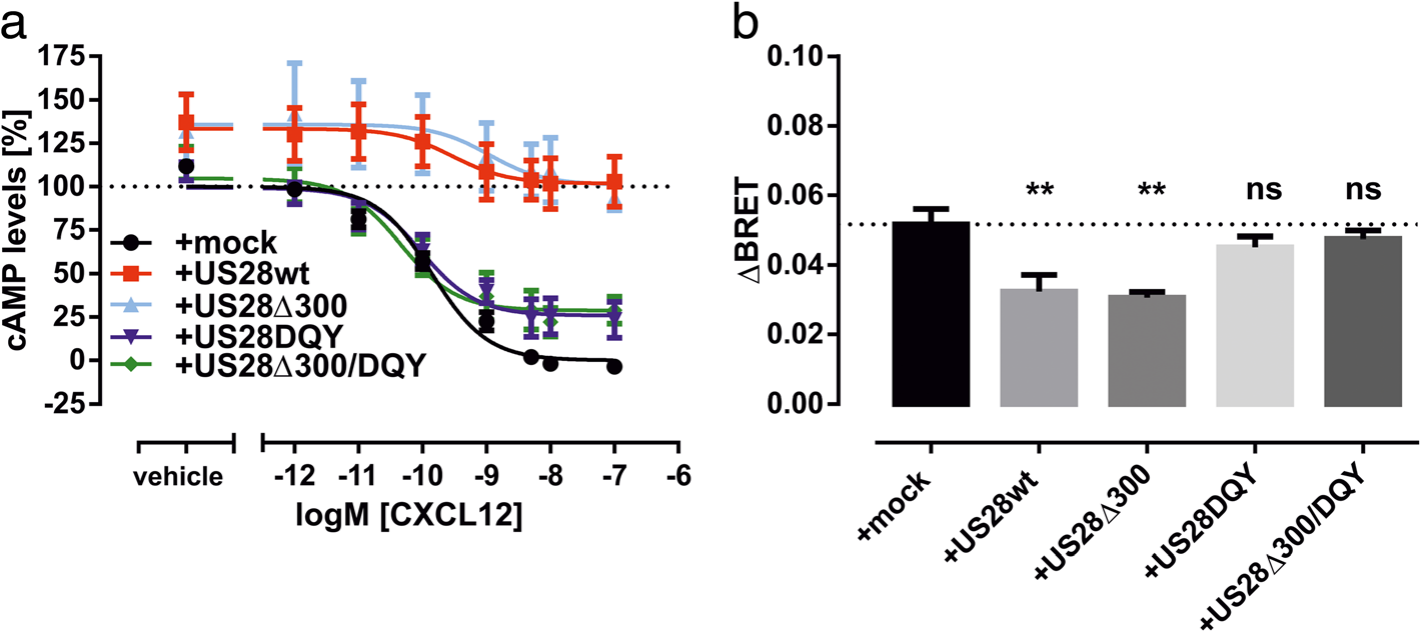
The dampening effect of US28 expression on CXCR4 - induced G protein-dependent signaling is controlled by the DRY motif. **a** Changes in agonist-induced cAMP concentrations after activation of CXCR4 were monitored in presence or absence of US28wt or US28 mutants. Cells were treated with CXCL12 at indicated concentrations and 10 μM forskolin for 15 min before measurement. BRET ratios were normalized on signal from mock-cotransfected cells stimulated with 100 nM CXCL12 (0%) or vehicle (100%). Curves represent means ± SEM of at least three independent experiments performed in triplicates (*n* = 3–8). **b** Agonist-induced recruitment of Gα_i1_ to CXCR4 in presence or absence of US28 or US28 mutants. BRET was measured in HEK293T cells cotransfected with CXCR4-Rluc8, Gα_i1_-91mVenus, Gβ1, Gγ2 and mock, US28wt or US28 mutants 2 min after addition of 100 nM CXCL12 or vehicle. ΔBRET was calculated by subtracting BRET ratios of vehicle-treated cells from BRET ratios of cells treated with 100 nM CXCL12 for each individual transfection. Columns represent means ± SEM of three independent experiments performed in quadruplicates. Statistical analysis was performed using one-way ANOVA with Dunnett’s post hoc test. *P < 0.05; **P < 0.01; ***P < 0.001; ns, not significant

CXCR4 is a Gα_i/o_ protein-specific receptor [22]. Upon binding and activation of CXCR4 by its endogenous ligand CXCL12, G_i/o_ proteins are activated, which results in an inhibition of adenylate cyclase (AC) and subsequent reduction of intracellular cAMP levels. On the contrary, US28 promiscously couples to different G protein subtypes from the Gα_q/11_, Gα_i/o_ Gα_s_ and Gα_12/13_ subfamilies [23–27]. US28 not only binds to several chemokines like e.g., RANTES (CCL5), MCP-1 (CCL2) or Fractalkine (CX3CL1) with high affinity [7, 23, 28], but is also highly constitutively active [28]. In order to assess the effect of US28 expression on the CXCL12-induced Gα_i/o_ protein-dependent signaling of CXCR4, we monitored the changes in cAMP levels by use of the BRET-based cAMP sensor CAMYEL. This biosensor is comprised of a catalytically inactive Epac1 that is fused to Citrine at its N-terminus and to *Renilla reniformis* luciferase (Rluc) at the C-terminus [29]. Binding of cAMP to CAMYEL results in a conformational change in the Epac1, which causes a decrease of BRET signal. In this way we determined the basal and CXCL12-induced changes in cAMP levels in presence and absence of US28. To assess the influence of the constitutive activity of US28 on CXCR4 signaling we included signaling-impaired mutants of US28 (US28Δ300, US28DQY and US28Δ300/DQY) in the assay. The US28DQY mutant possesses a mutation R129Q that disrupts the DRY motif. This leads to a loss of constitutive G protein activation [30]. The US28Δ300 mutant carries a truncated C-terminus (the last 54 amino acids including important serine and threonine residues were removed) and shows slower constitutive endocytosis rates and increased constitutive G protein signaling [30, 31]. The double mutant US28Δ300/DQY combines both of these phenotypes. For the assay HEK293T cells were transiently transfected with CXCR4 and CAMYEL and stimulated with endogenous chemokine ligand CXCL12. CXCL12 dose-dependently decreased cAMP levels with a subnanomolar IC_50_ (Fig. 1a; Table 1). Coexpression of US28wt or US28Δ300 with CXCR4 significantly abated CXCL12-induced decrease in cAMP levels to about 35% of the absolute efficacy observed in mock-cotransfected cells (Fig. 1a; Table 1). Moreover, the presence of US28wt or US28Δ300 induced higher basal cAMP levels (Fig. 1a). As evident from Additional file 1: Figure S1a, CXCR4 does not display constitutive Gα_i_ protein activation as the basal level of cAMP in CXCR4-expressing cells was not different from CAMYEL-sensor only expressing cells. In contrast, basal cAMP levels in US28 expressing cells were increased to the same level as observed for US28/CXCR4 coexpressing cells. The G protein-uncoupled mutants US28DQY and US28Δ300/DQY restored up to 75% of the agonist-induced decrease in cAMP levels (Fig. 1a; Table 1) and did not induce higher basal levels of cAMP. As the effect of US28Δ300 on CXCR4 signaling was comparable to US28wt and the double mutant US28Δ300/DQY did not behave differently from US28DQY, the C-terminal domain of US28 does not seem to be involved in modulation of G protein-mediated signaling of CXCR4.

**Table 1:** Influence of US28 coexpression on efficacy and potency of CXCL12-induced cAMP concentrations

Cotransfection	E_max_ (mean ± SEM)	pEC_50_ (mean ± SEM)	*n*
CXCR4 + mock (control)	100 ± 4	9.85 ± 0.09	8
CXCR4 + US28	32 ± 11 (***)	9.54 ± 0.91 (n.s.)	4
CXCR4 + US28Δ300	35 ± 18 (***)	8.96 ± 1.10 (n.s.)	3
CXCR4 + US28DQY	73 ± 7 (n.s.)	10.02 ± 0.22 (n.s.)	5
CXCR4 + US28Δ300/DQY	76 ± 8 (n.s.)	10.34 ± 0.26 (n.s.)	4

Data shown correspond to Fig. 1a. Efficacy (E_max_) is calculated as the absolute value of the maximal CXCL12-induced effect normalized on CXCR4-only expressing cells (100%). Potency is displayed as pEC_50_. Data were derived from three to eight independent experiments each performed in triplicate wells and are presented as mean ± SEM. Statistical analysis was performed using one-way ANOVA with Dunnett’s post hoc test comparing US28 coexpressing systems with CXCR4-only expressing cells (control). ***P < 0.001,* n.s*. not significant

### US28 affects interactions between G_i_ proteins and CXCR4

We also determined the effect of US28 expression on the G protein-dependent signaling of CXCR4 as early as on the level of Gα_i1_ protein recruitment. Therefore, we again used a BRET-based method. Gα_i1_-91mVenus and unlabeled Gβ_1_ and Gγ_2_ were coexpressed with CXCR4 carrying a *Renilla reniformis* luciferase 8 (Rluc8) at its C-terminus [32]. The agonist-induced recruitment of the Gα_i1_ subunit to the chemokine receptors was monitored in presence and absence of US28wt or US28 mutants. In CXCR4-expressing HEK293T cells, stimulation with 100 nM CXCL12 resulted in a significant increase in ligand-promoted BRET signal (ΔBRET). In US28wt or US28Δ300 coexpressing cells Gα_i1_ recruitment to CXCR4 was significantly reduced in efficacy, whereas the coexpression of US28DQY or US28Δ300/DQY did not significantly suppress the agonist-induced Gα_i1_ protein recruitment to CXCR4 (Fig. 1b). As evident from Additional file 1: Figure S1b, the maximal Gα_i1_ recruitment to CXCR4 in presence of US28 does not increase with an increasing pool of Gα_i1_ proteins, indicating that the recruitment of Gα_i1_ proteins is not influenced by a limited pool of Gα_i1_ proteins.

### US28 restrains surface expression and CXCL12-mediated β-arrestin 2 recruitment to CXCR4

**Fig. 2:**
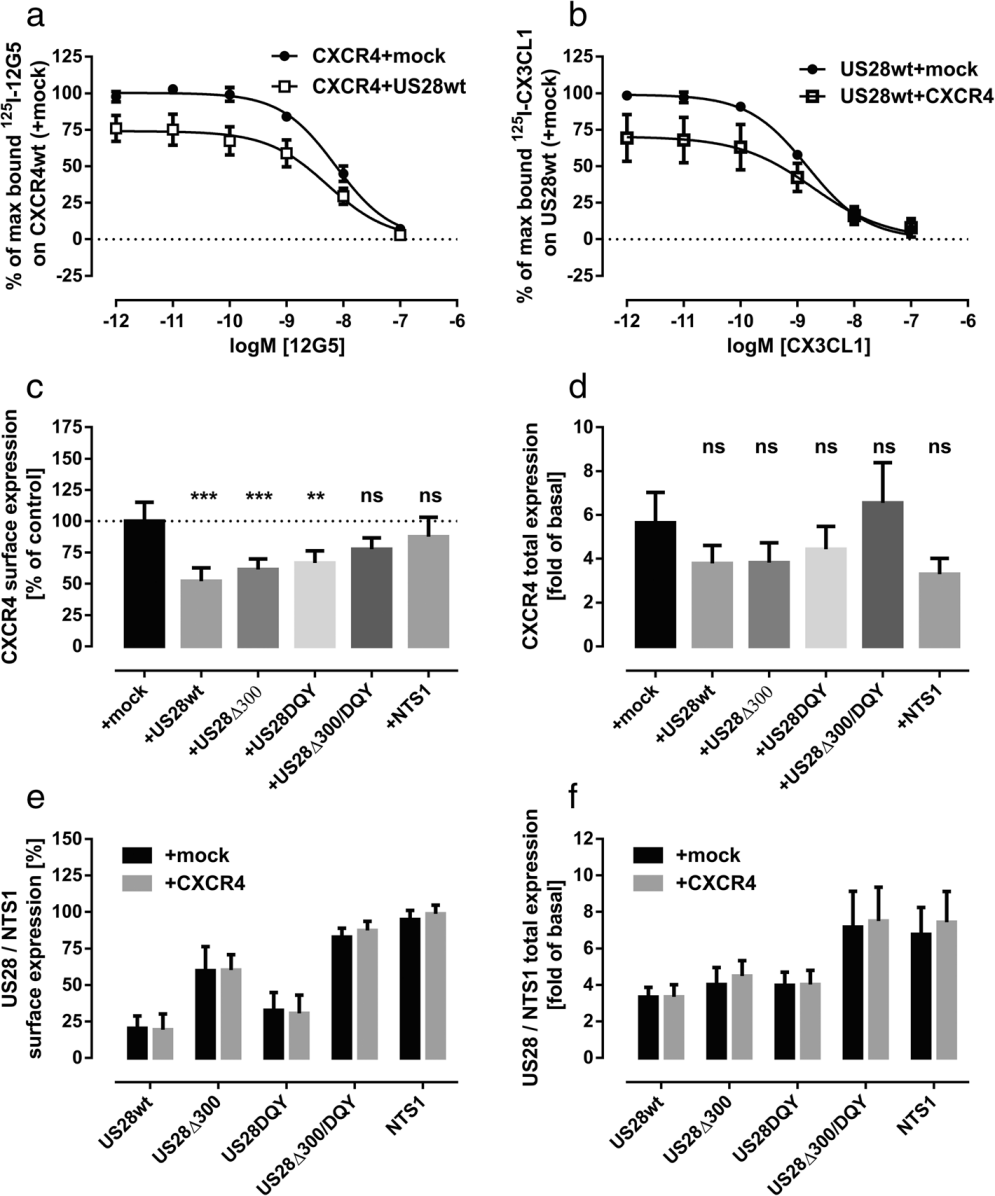
Analysis of CXCR4 and US28 total and surface expression in mono-and coexpressing cells. **a**, **b** Radioligand-displacement studies to detect changes in CXCR4 and US28 surface expression were performed in transiently transfected COS-7 cells. Dose response curves represent the means ± SEM of seven independent experiments, each performed in duplicate. **c**-**f** For the ELISA - based analysis N-terminally FLAG-tagged CXCR4 and N-terminally HA-tagged US28, US28mutants and NTS1 were expressed in HEK293T cells. **c** Surface expression of CXCR4 was calculated as the signal ratio between permeabilized and non-permeabilized cells (reflected by FLAG-immunoreactivity) and normalized on the surface expression in CXCR4-only expressing cells. **d** The total expression of CXCR4 in mono- and coexpressing cells was calculated as a factor of FLAG-immunoreactivity in mock-transfected cells. **e** Surface expression of US28, US28 mutants and NTS1 in presence and absence of CXCR4 was calculated as the signal ratio between permeabilized and non-permeabilized cells (reflected by HA-immunoreactivity). **f** The total expression of US28, US28 mutants and NTS1 in presence and absence of CXCR4 was calculated as a factor of HA-immunoreactivity detected in mock-transfected cells. Columns represent means ± SEM from at least three independent experiments (*n* = 3–5), each performed in triplicates. Statistical analysis was performed using one-way ANOVA with Dunnett’s post hoc test. (*P < 0.05; **P < 0.01; ***P < 0.001; ns, not significant)

A reduction of surface expression of chemokine receptors CXCR4 as well as CCR1, CCR2 and CCR5 was observed in monocytes upon their infection with endotheliotropic strains (TB40E and VHLE) and clinical isolates of HCMV [33]. This downregulation was attributed to a changed distribution between the cytoplasm and the cell membrane. Also the coexpression of UL33 and UL78 vGPCRs encoded by HCMV was shown to lead to altered surface expression of chemokine receptors like CCR5 and CXCR4 [3]. As the reduced efficacy but not potency of G protein-dependent signaling of CXCR4 in presence of US28 could reflect a reduced number of available receptors on the cell surface, we determined the effect of US28 on total and surface expression of CXCR4. We initially used a whole-cell radioligand-binding assay using either [^125^I]12G5 to detect the surface expressed CXCR4 or [^125^I]CX3CL1/Fractalkine to detect surface-expressed US28. The data from this radioligand binding assay in COS-7 cells showed that the coexpression of US28 with CXCR4 resulted in reduced binding of the radioligand without any effect on pK_i_ values (Table 2). This is indicative of a reduced number of receptors expressed on the cell surface. The effect was reciprocal – the expression of US28 resulted in a reduced expression of CXCR4 and CXCR4 caused the reduced expression of US28 on the surface (Fig. 2a, b). To confirm these observations, we performed an enzyme-linked immunosorbent assay (ELISA). With this assay we compared the influence of wild type US28 and its signaling impaired mutants on the total and surface expression of CXCR4. In the same assay the change in US28 expression and cellular distribution were monitored. The neurotensin receptor type 1 (NTS1) was used as a negative control for possible artifacts caused by transient expression of receptors. CXCR4 was FLAG-tagged, the US28 mutants and NTS1 were HA-tagged at the N-terminus and transiently expressed in HEK293T cells at given combinations (Fig. 2c-f). To differentiate between the surface and total expression, one set of probes was permeabilized with TritonX-100 to detect the total number of expressed receptors. Total expression of CXCR4 was not significantly changed in all coexpression systems as analyzed by one-way ANOVA with Dunnett’s post hoc test comparing coexpression systems with mock-transfection (Fig. 2d). As evident from Fig. 2c, coexpression of US28wt reduced the surface expression of CXCR4 by up to 50%. Coexpression with US28DQY or US28Δ300 still significantly reduced the surface expression of CXCR4. Only when the double mutant US28Δ300/DQY was coexpressed, CXCR4 surface expression was not significantly altered, indicating that the constitutive activity as well as the C-terminus of US28 substitutably contribute to downregulation of CXCR4 steady-state surface expression levels. Importantly, coexpression with NTS1 did not influence CXCR4 surface expression, indicating that the observed reduction in CXCR4 surface expression is not an artifact of transient transfection but a direct result of US28 coexpression. Additionally, we included US27, another HCMV - encoded vGPCR, as a control. US27 is found predominantly in perinuclear vesicles like US28 [34]. Moreover, US27 was described to upregulate CXCR4 signaling as a result of increased CXCR4 protein expression levels [35]. We could reproduce this upregulation of total CXCR4 expression levels (Additional file 2: Figure S2). In contrast to US28, US27 does not alter the distribution of CXCR4 between plasma membrane and cytoplasm. This further underlines the hypothesis that the HCMV - encoded vGPCRs interfere with the chemokine receptor system in multiple ways. In contrast to data from the whole-cell radioligand-binding assay, we found the total and surface expression of US28wt and mutants to be unaltered in the presence of CXCR4 (Fig. 2e, f). As a result of slower recycling rates the US28Δ300 and US28Δ300/DQY were expressed on the cell surface to a higher extent than US28wt and US28DQY, as described before [36].

**Table 2:** Radioligand-displacement studies to detect changes in CXCR4 and US28 surface expression

Cotransfection	Used Radioligand	% of max. bound radioligand (mean ± SEM)	pK_i_ (mean ± SEM)	*n*
CXCR4 + mock (control)	[^125^I]12G5	100 ± 2	8.13 ± 0.07	7
CXCR4 + US28wt	[^125^I]12G5	74 ± 5 (***)	8.27 ± 0.26 (n.s.)	7
US28wt + mock (control)	[^125^I]CX3CL1	99 ± 3	8.82 ± 0.08	7
US28wt + CXCR4	[^125^I]CX3CL1	70 ± 10 (*)	8.71 ± 0.43 (n.s.)	7

Data presented are derived from seven independent experiments, each performed in duplicate wells, presented as mean ± SEM. Statistical analysis was performed using Student’s *t* test, comparing coexpression with mock-cotransfection (control). *P < 0.05; ***P < 0.001, *n.s.* not significant

**Fig. 3:**
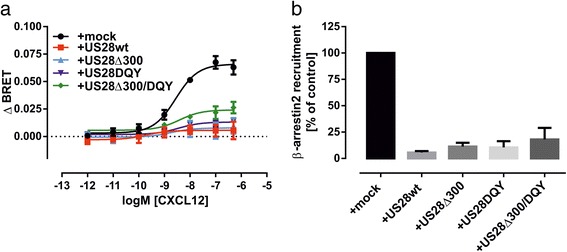
Agonist-induced β-arrestin 2 recruitment to CXCR4 in presence and absence of US28wt and mutants. **a** BRET-based approach. CXCR4 C-terminally tagged with mVenus, β-arrestin 2 N-terminally fused to RlucII and US28, US28 mutants or empty vector (mock) were transiently expressed in HEK293T cells. β-arrestin 2 recruitment was measured 5 min post-ligand addition. ΔBRET was calculated by subtracting BRET ratio detected in vehicle stimulated cells from BRET ratio detected in CXCL12-stiumulated cells for each receptor-combination. Curves represent means ± SEM from at least three independent experiments (*n =* 3–6), each performed in triplicates. **b** BiLC-based approach. CXCR4 C-terminally tagged with ElucC, β-arrestin2 N-terminally fused to ElucN and US28, US28 mutants or empty vector (mock) were transiently expressed in HEK293T cells. Luminescence was measured following 10 min stimulation with 100nM CXCL12 or vehicle (no filters, 2 s recording). ΔLuminescence was calculated by subtracting luminescence detected in vehicle stimulated cells from luminescence detected in cells stimulated with 100nM CXCL12 for each transfection-combination and normalized on ΔLuminescence of mock-cotransfected cells. Columns represent means ± SEM from three independent experiments, each performed in triplicates

As phosphorylation by G protein-coupled receptor kinases (GRKs) and subsequent recruitment of β-arrestins are employed by CXCR4 as a mechanism of desensitization, we also investigated the influence of US28wt and mutants on agonist-induced β-arrestin 2 recruitment to CXCR4. For this assay we used a β-arrestin 2 construct carrying *Renilla reniformis* luciferase 2 (RlucII) at the N-terminus and CXCR4 carrying mVenus at its C-terminus. We then monitored the β-arrestin 2 recruitment to CXCR4 in presence and absence of US28wt and mutants. The coexpression of US28wt with CXCR4 almost completely abolished agonist-induced β-arrestin 2 recruitment to CXCR4. The coexpression of mutants US28Δ300 or US28DQY did not significantly restore the β-arrestin 2 recruitment to CXCR4 (Fig. 3a). Only the coexpression with US28Δ300/DQY led to partial restoration of agonist-induced β-arrestin 2 recruitment to CXCR4.

To validate our data obtained in the BRET-based β-arrestin 2 recruitment assay, we employed a bioluminescence complementation (BiLC)-based system developed by Ozawa et al. [37, 38]. They optimized the complementation of split luciferase fragments from click beetle (Brazilian *pyrearinus termitilluminans*) to provide a BiLC-system with high sensitivity and low signal-to-noise ratio. As shown in Fig. 3b, the data obtained from BiLC-based β-arrestin 2 recruitment are comparable to the data from the BRET-based approach. Additionally, we verified that the attenuation of β-arrestin 2 recruitment to CXCR4 in the presence of US28wt is not caused by a limited pool of β-arrestin 2 available for the interaction. As shown in Additional file 3: Figure S3, the reduction of β-arrestin 2 recruitment to CXCR4 in presence of US28wt is not influenced by an increasing pool of β-arrestin 2.

### CXCR4 does not heteromerize with US28

Among GPCR researchers there is an ongoing debate about the existence and importance of GPCR dimers in vivo. However, several reports showed the organization of class A GPCRs in homodimeric, oligomeric or even heteromeric complexes [21, 39–41]. Methods such as pull-down assays, protein crystallography, BRET, fluorescence resonance energy transfer (FRET), fluorescence recovery after photobleaching (FRAP) and single molecule imaging provide tools to track GPCR dimerization in living cells [42–45]. Also bitopic ligands are important tools to analyze and manipulate receptor dimerization [46].

**Fig. 4:**
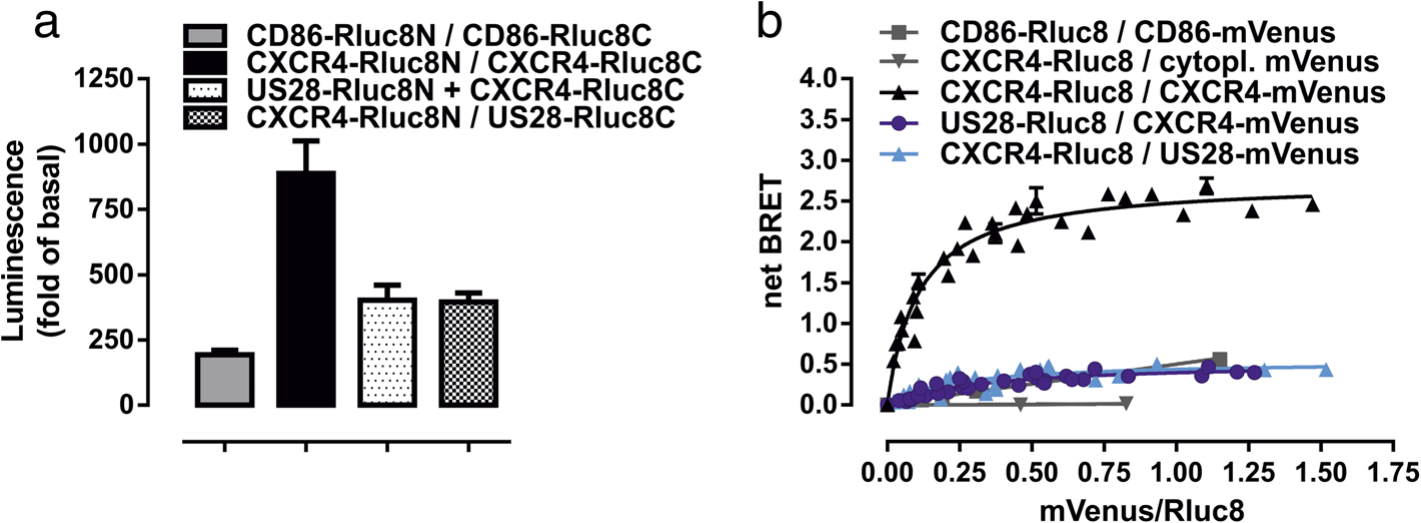
Analysis of heteromerization between CXCR4 and US28 using BRET and BiLC. **a** BiLC using protomers of Rluc8 (Rluc8N/Rluc8C) to assess receptor dimerization. Columns show the factor of Rluc8 activity measured in mock-transfected cells. Columns represent means ± SEM of at least three independent experiments, each performed in triplicates. **b** BRET donor saturation curves by cotransfecting a fixed amount of receptor-Rluc8 in presence of increasing amounts of receptor-mVenus constructs. net BRET was calculated by subtracting BRET ratio of donor-only expressing cells. Curves represent pooled net BRET ratios (±SD) from three independent experiments, each performed in quadruplicates. Curves were fitted using least square nonlinear regressions assuming a one site hyperbola. Data from negative controls were additionally fitted using linear regression

Initially, we used BiLC-technology to study dimerization of CXCR4 and US28. For this purpose, we created necessary expression vectors for C-terminally tagged receptors using previously described splits of *Renilla reniformis* luciferase 8 (Rluc8) [31]. As a control for nonspecific interactions, we used CD86, which is known to behave as a monomer and is routinely used as a monomeric control protein in studies of e.g., dimerization of GPCRs [43, 47, 48]. As expected, the strongest signal was obtained in the case of CXCR4 homodimers, which is in accordance with the literature reporting homodimerization and oligomerization of CXCR4 [17, 49]. The luminescence intensity at corresponding heterodimers indicated the presence of weak heteromerization for US28-CXCR4 (Fig. 4a). To estimate the reliability of our results, we also used two similar BiLC-based approaches based on splits of *firefly* and *emerald* luciferase [37]. As expected, the two additional BiLC-based systems yielded comparable results (Additional file 4: Figure S4). Because results from BiLC are highly dependent on cell numbers and expression levels, we further validated our observations by a BRET-based assay, which enables differentiation between the real dimerization and the false positive signal due to random collision [44]. A constant amount of donor-labeled protein is coexpressed with increasing amounts of acceptor-labeled protein [39]. By plotting BRET ratios as a function of acceptor/donor expression levels the specific signal can be distinguished from unspecific signal. While a specific interaction is known to result in saturation of the BRET signal, a nonspecific interaction yields a quasi-linearly increasing BRET signal. For our experiments receptors were C-terminally tagged with Rluc8 or mVenus. Donor saturation curves were obtained by cotransfecting a fixed DNA amount of receptor-RLuc8 in the presence of increasing amounts of receptor-mVenus. As negative controls, a homomeric pair of CD86 with the corresponding tags, as well as cytoplasmic mVenus was used. A hyperbolic donor saturation curve reaching an asymptote with increasing mVenus/RLuc8 ratios was clearly observed for CXCR4 homodimers (Fig. 4b). For the negative controls, a linear increase in net BRET was detected with increasing mVenus/RLuc8 ratios, reflecting a nonspecific interaction because of linearly increasing random collision. Low maximal BRET (BRET_max_) and high BRET_50_ values indicate low probability for physical interactions between US28 and CXCR4 (Table 3). Moreover, the orientation of BRET sensors did not influence this result, as the reciprocally tagged pair of US28-CXCR4 yielded comparable results.

**Table 3:** BRET_max_ and BRET_50_ values (mean ± SEM) from BRET donor saturation curves to detect receptor dimerization

Cotransfection	BRET_max_ (mean ± SEM)	BRET_50_ (mean ± SEM)	*n*
CXCR4-Rluc8/CXCR4-mVenus	2.75 ± 0.042	0.11 ± 0.007	3
CXCR4-Rluc8/US28-mVenus	0.54 ± 0.031	0.24 ± 0.036	3
US28-Rluc8/CXCR4-mVenus	0.53 ± 0.023	0.33 ± 0.031	3
CD86-Rluc8/CD86-mVenus	n.d.	n.d.	3
CXCR4-Rluc8/cytopl. mVenus	n.d.	n.d.	3

Curves were fitted using nonlinear regression assuming a one-site hyperbola

Values are derived from pooled netBRET ± SD from three independent experiments, each performed in quadruplicates

Legend: *n.d.* not to be determined

**Fig. 5:**
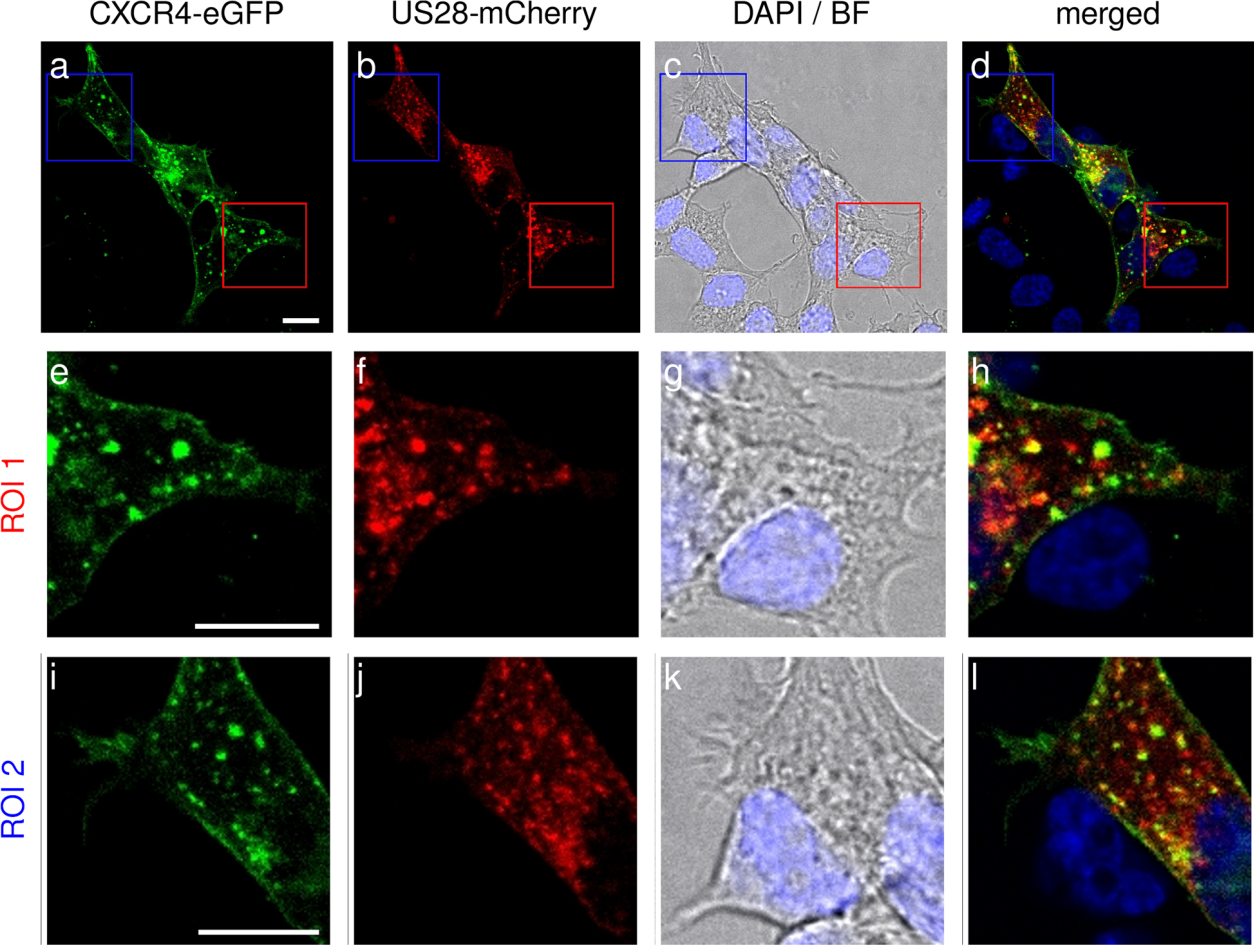
Qualitative colocalization studies using confocal laser scanning microscopy. CXCR4 C-terminally fused to eGFP and US28 C-terminally tagged with mCherry were coexpressed in HEK293T cells. Insets ROI 1 (**e**-**h**) and ROI 2 (**i**-**l**) show magnifications of the indicated areas in panels (**a**-**d**). Cell nuclei were stained with DAPI. Scale bars in panels (**a**), (**e**) and (**i**) and represent 10 μm

In order to additionally assess the subcellular localization and colocalization of CXCR4 and US28 we investigated transiently expressing C-terminally labeled receptors (either with mCherry or eGFP) in HEK293T cells. As evident from Fig. 5, CXCR4 is expressed mainly on the cell surface whereas in turn US28 is found in intracellular vesicles. Colocalization was weak and restricted to intracellular vesicles.

Overall, BiLC and BRET results demonstrated that US28 most likely does not form heterodimers with CXCR4. Considering that also images from confocal laser scanning microscopy showed that CXCR4 and US28 only weakly colocalize and that this colocalization is restricted to intracellular vesicles, we showed that US28 does not influence CXCR4 signaling by heteromerization. The localization of US28 in endosomes implies that the effect of US28 on CXCR4 is ligand-independent.

### US28 is involved in downregulation of CXCR4 in HCMV-infected HUVEC

**Fig. 6:**
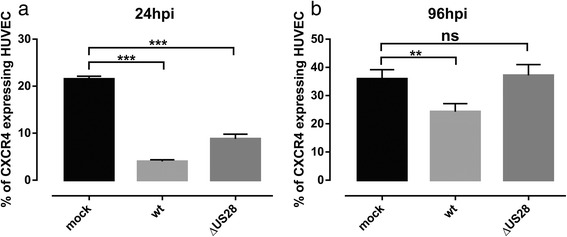
CXCR4 surface expression in infected HUVEC. At 24 and 96 hours post infection (hpi), mock- and TB40E/IE2eYFP- (wt) or TB40E/IE2eYFP-delUS28- (ΔUS28) infected HUVEC were examined by FACS for surface expression of CXCR4. Fluorescently labeled IE2-eYFP enabled detection of CXCR4 surface expression in lytically-infected cells only. The percentages of HUVEC expressing CXCR4 were evaluated in mock- or lytically-infected (IE2-positive) cells at 24 hpi (**a**) or 96 hpi (**b**). Values are the mean ± SD of three experiments

As we observed that the presence of US28 caused a strong downregulation of CXCR4 from the cell surface in HEK293T cells, which is connected with a drastic loss of signaling ability, we next analyzed this interaction in the viral context. Therefore, we constructed a recombinant virus TB40E/IE2eYFP-delUS28 which lacks the US28 gene and possesses an eYFP-tagged IE2 enabling detection of lytically-infected cells. The TB40E/IE2eYFP-delUS28 and the previously described TB40E/IE2eYFP [50] viruses allowed us to monitor CXCR4 surface expression in lytically-infected cells via Fluorescence-activated cell sorting (FACS) analysis. We infected primary human umbilical vein endothelial cells (HUVEC) at a multiplicity of infection (MOI) of 2 which yielded between 10 and 30% lytically-infected cells. We then monitored CXCR4 surface expression in mock- and lytically-infected cells at early (24 hpi) and late (96 hpi) time points of infection. At 24 hpi, TB40E/IE2eYFP- and TB40E/IE2eYFP-delUS28-infected HUVEC showed strongly downregulated CXCR4 surface expression (Fig. 6a). Strikingly, at 96 hpi CXCR4 surface expression was significantly downregulated in TB40E/IE2eYFP-infected HUVEC, whereas in TB40/IE2eYFP-delUS28-infected HUVEC CXCR4 surface expression was restored to mock level (Fig. 6b).

## Discussion

In this study we show that expression of the cytomegaloviral chemokine receptor US28 leads to downregulation of CXCR4 surface expression and agonist-induced signaling in HEK293T cells. These findings are in accordance with the observation that in primary HUVEC, infected with the endotheliotropic TB40E strain of HCMV, CXCR4 is significantly downregulated from the surface of infected cells. In contrast, using a TB40E strain lacking the US28 gene we detected that CXCR4 downregulation is strongly impaired, in particular at late times after infection indicating an important role of US28 for CXCR4 modulation during the course of HCMV infection.

We assessed the consequences of US28 expression for the responsiveness of CXCR4 and could narrow down the underlying mechanism to structural motifs of US28. Our data demonstrate that the presence of US28 in CXCR4-expressing cells leads to a dampening of CXCL12-induced Gα_i_ protein-dependent signaling. This inhibition could be observed as early as on the level of Gα_i_ protein recruitment as well as on the level of the secondary messenger cAMP. Additionally, US28 seems to antagonize the ligand-induced Gα_i1_ recruitment of CXCR4 by its constitutive G protein activation. The G protein-uncoupled mutants US28DQY and US28Δ300/DQY had no significant effect on agonist-induced second messenger formation and Gα_i_ protein recruitment of CXCR4, clearly showing that mainly the DRY motif, enabling the high constitutive activity of US28, is responsible for dampening of the G protein-dependent signaling of CXCR4, while the C-terminal domain does not seem to play a role.

The constitutive signaling activity of US28 attracted attention before and is suspected to represent one of the mechanisms employed by vGPCRs to disturb the host immune homeostasis [28, 51]. US28 was reported to constitutively activate phospholipase C-β (PLC-β) and NFκB via G_q/11_-dependent pathways [23, 52]. Infection with US28R129A mutant virus failed to induce PLC-β signaling, which also shows the clinical relevance of constitutive G protein activation by US28 [53]. Moreover, US28-mediated constitutive G protein activation is also involved in tumor formation and progression [54]. The C-terminally truncated forms of US28, lacking important serine and threonine residues, are expressed to a higher degree on the cell surface than US28wt and therefore show even higher G protein-dependent constitutive activity than wild type US28 [30]. This also explains the effect of the US28Δ300 mutant on Gα_i_ protein-dependent signaling of CXCR4 being comparable to US28wt.

Interestingly, in presence of US28wt, US28Δ300 and US28DQY the CXCL12-induced β-arrestin 2 recruitment to CXCR4 was abrogated. Only the concomitant expression of US28Δ300/DQY led to partial recovery of the initial agonist-induced β-arrestin 2 recruitment to CXCR4. This indicates that the constitutive G protein activation as well as the C-terminal phosphorylation sites of US28 are involved in US28-promoted abrogation of β-arrestin 2 recruitment to CXCR4, but are not the main determinants. Recently, it was shown that activation of ERK1/2 leads to a β-arrestin 2-dependent reduction of constitutive GPCR cell surface expression and consequently blunted G protein and β-arrestin signaling [55]. US28 was reported to activate ERK1/2 in an agonist-dependent manner, engaging the G proteins Gα_i1_ and Gα_16_, in response to RANTES/CCL5 [25]. As described before, also an agonist-independent downstream activation of ERK1/2 can be observed in US28-expressing HEK293T cells [56]. With use of the MEK1/2 inhibitor PD184352 and the ERK1/2 inhibitor FR180204 we intended to suppress US28-mediated ERK1/2 activation and thus reduction of CXCR4 surface expression. However, this experiment did not reveal significant changes in steady-state surface expression levels (data not shown). Consequently, in case of US28, the weak constitutive activation of ERK1/2 via the G_q/11_ pathway does not act as the main determinant leading to the radical dampening of CXCR4 signaling. US28 itself was shown to employ multiple routes for internalization including dynamin-dependent pathways. However, US28 trafficking is not dependent on β-arrestin, as in β-arrestin deficient cells endocytosis and subcellular localization of US28 was unaltered [57]. Nevertheless, the presence of US28 in HEK293T cells was shown to cause a redistribution of β-arrestin 2 from the plasma membrane to intracellular vesicles in absence of ligand stimulation [58]. In contrast to the G protein-uncoupled mutant US28R129A, a GRK phosphorylation site-deficient mutant of US28, US28S1-12A [59], showed the same effect on subcellular β-arrestin 2 localization. This indicates that the DRY motif, conserved in TM3 of US28 and responsible for constitutive G protein activation, is sufficient to cause a redistribution of β-arrestin 2 to intracellular vesicles, which reduces its availability to interact with other receptors. Still, the signaling-deficient US28DQY was not sufficient to prevent abrogation of agonist-induced β-arrestin 2 recruitment to CXCR4 in our hands. Therefore, the mechanism of US28-mediated abrogation of agonist-induced β-arrestin 2 recruitment to CXCR4 remains to be unraveled.

As heteromerization is one of the mechanisms that enables receptors to influence and disturb each other’s signaling we also thoroughly investigated the possibility of CXCR4/US28 heteromerization. However, our data indicate that the observed dampening of CXCR4 responsiveness by US28 cannot be explained by receptor heteromerization. Data from BiLC and BRET saturation experiments suggest a weak, most probably non-significant interaction between CXCR4 and US28. Furthermore, qualitative analyses of images from colocalization studies using confocal laser scanning microscopy show that CXCR4 does not colocalize with US28 on the cell surface and intracellular colocalization was confined to single vesicles.

We hypothesize that the attenuation of G protein- and β-arrestin 2-dependent signaling of CXCR4 is related to a reduced density of CXCR4 at the cell surface as we found the surface expression of CXCR4 to be downregulated for up to 50% in the presence of US28wt in HEK293T cells. We observed that coexpression of the signaling-impaired mutants US28Δ300 and US28DQY still significantly reduced CXCR4 surface expression. Only when the double mutant US28Δ300/DQY was coexpressed, CXCR4 surface expression was restored. This indicates that only when the constitutive G protein signaling and the recycling-machinery of US28 are impaired at the same time CXCR4 surface expression is not attenuated. However, we could also show that not only in HCMV strain TB40E-infected monocytes [33], but also in infected HUVEC CXCR4 surface expression is significantly attenuated, which underlines the relevance of our study. We observed that TB40E/IE2eYFP- and TB40E/IE2eYFP-delUS28-infected HUVEC show strong downregulation of CXCR4 surface expression at 24 hpi. At late time points of infection (96 hpi) CXCR4 surface expression was significantly downregulated in TB40E/IE2eYFP-infected HUVEC, whereas in TB40E/IE2eYFP-delUS28-infected HUVEC, CXCR4 surface expression was restored to mock level. This indicates that US28 a critical factor involved in attenuation of CXCR4 surface expression in particular at late time points of infection, which also correlates with the late expression kinetic of US28 [60]. The observed downregulation of CXCR4 at early time points of infection is most probably attributed to other factors. However, downregulation of chemokine receptors in infected monocytes eventually impaired immune response to viral infection as shown by Frascaroli et al. [33]. HCMV - infected monocytes failed to recruit lymphocytes, monocytes and neutrophils as a result of downregulated CCR1, CCR2, CCR5 and CXCR4 levels at the cell surface. Endothelial cells (EC) are described to play a role in the dissemination of HCMV throughout the body [61]. Interestingly, during acute disease EC can detach from the blood vessel and enter the blood stream [62]. In contrast to detection of HCMV infected EC during acute infection in immunocompromised patients, their role during latency is controversial. There are reports demonstrating HCMV DNA in vessel walls of major arteries of sero-positive individuals [63], whereas others classify EC as unlikely sites of HCMV latency in vivo [64]. Downregulation of chemokine receptors from the surface of EC might facilitate detachment of EC from the blood vessel and entry into the blood stream, thereby facilitating viral dissemination. In accordance with reports about US28 being directly involved in facilitation of viral spread [60, 65], we propose that US28 might also indirectly promote viral dissemination by downregulation of chemokine receptors from the surface of infected cells.

## Conclusion

In summary, our data support the assumption that the observed attenuation of CXCR4 surface expression and signaling in presence of US28 is mainly caused by the high constitutive activity of US28. By use of well-characterized mutants of US28, we could attribute the reduction of G protein-dependent signaling and surface expression of CXCR4 to an activity relying partially on the DRY motif and C-terminus of US28. We propose that the constitutive signaling of US28 leads to indirect signaling crosstalk via shared intracellular signaling networks. This eventually results in disturbed chemokine receptor signaling and reduced constitutive surface expression, which is also reflected in HCMV-infected primary HUVEC.

## Methods

### Cell culture and transfection

Human Embryonic Kidney 293 (HEK293) T cells were cultured in DMEM/F-12 supplemented with 10% (vol/vol) fetal bovine serum (FBS), 1% penicillin-streptomycin, 2 mM L-glutamine and incubated at 37 °C/5% CO_2_. Transient transfections were performed using linear polyethylenimine 25 kDa (PEI) (Polysciences, Inc.) or TransIT-293 transfection reagent (Mirus corporation) as transfection reagent at a transfection reagent/DNA ratio of 3:1. COS-7 cells were grown in Dulbecco’s modified Eagle’s medium 1885 supplemented with 10% FBS, 2 mM glutamine, 180 units/ml penicillin and 45 μg/ml streptomycin at 37 °C/10% CO_2_. Primary human foreskin fibroblasts (HFFs) were prepared from human foreskin tissue [66] and cultured in Eagle’s minimal essential medium supplemented with 7.5% FBS, 1% L-glutamine and gentamicin at 37 °C/5% CO_2_. Primary HUVEC (a kind gift from M. Mach, Erlangen, Germany) were isolated from single blood veins from human umbilical cord tissue and cultured in Endothelial Growth Medium supplemented with 5% FBS, hydrocortisone, human Fibroblast Growth Factor B (hFGF-B), Vascular Endothelial Growth Factor (VEGF), human insulin-like growth-factor-I (R3-IGF-1), ascorbic acid, human epidermal growth factor (hEGF) and GA-1000 (Gentamicin, Amphotericin B) at 37 °C/5% CO_2_.

### Virus infection

Infection experiments were performed with the recombinant viruses TB40E/IE2-eYFP [50] and TB40E/IE2eYFP-delUS28. Titration of the viral stocks was performed by IE1p72 fluorescence [67]. Briefly, HFFs (8 × 10^4^ cells) in 0.5 ml medium were seeded into 24-well plates and infected the next day with 300 μl of various dilutions (1:5 to 1:5^5^) of viral supernatant. At 2 hpi 500 μl of fresh culture medium were added. At 36 hpi, cells were fixed with 4% PFA and stained with monoclonal antibody p63-27, which is directed against IE1p72 [68]. Subsequently, the number of IE1-positive cells was determined in duplicate wells and was used to calculate viral titers in IE1 protein-forming units (IE1U) per ml. For infection, 2 × 10^5^ HUVEC, between passage two and seven, were seeded per well in 6-well plates. The day after, culture medium was replaced by 2 ml of infectious cell culture supernatant of TB40E/IE2-eYFP or TB40E/IE2-eYFP-delUS28 and the plates were centrifuged for 30 min at 1900 × *g*. After 3 h of incubation, the supernatant was substituted with fresh culture medium.

### Generation of the recombinant virus TB40E/IE2eYFP-delUS28

For generation of the recombinant virus TB40E/IE2eYFP-delUS28 the coding region of US28 was removed from the already described HCMV TB40E/IE2eYFP [50] by BAC (bacterial artificial chromosome) mutagenesis according to Datsenko & Wanner [69]. *E.coli* strain DH10B, which had beforehand been transformed with TB40E/IE2eYFP BAC DNA and pKD46 (Red recombinase expression plasmid with a temperature sensitive, L-arabinose inducible promoter) [69], were grown in LB medium supplemented with chloramphenicol, ampicillin and 0.2% L-arabinose at 30 °C. In order to accomplish homologous recombination *E. coli* cells were transformed with PCR fragments, generated by amplification of an FRT-kanamycin-FRT cassette from plasmid pKD13 [69] using primers that are homologous to the adjacent regions of the US28 gene. *DpnI* was added to digest template DNA and the amplicon was purified from an agarose gel. Positive transformants were identified using agar plates containing chloramphenicol and kanamycin at 37 °C and additionally checked for the clearance of the Red recombinase plasmid pKD46 by use of agar plates containing ampicillin. Subsequently, chloramphenicol/kanamycin-resistant, but ampicillin-sensitive clones were transformed with pcP20 in order to enable elimination of the kanamycin cassette. pcP20 encodes for a FLP recombinase expression plasmid, which is chloramphenicol/ampicillin-resistant and shows temperature-sensitive replication and thermal induction of FLP recombinase expression [70]. Chloramphenicol/ampicillin-resistant mutants were selected at 30 °C and then purified for pCP20 at 43 °C. Finally, chloramphenicol-resistant but ampicillin/kanamycin-sensitive transformants were selected at 37 °C. BAC DNA was isolated from bacteria and the obtained BACs were verified by distinct PCR reactions and subsequent sequencing as well as restriction fragment length polymorphism analysis (RFLP) as described previously [71]. In order to reconstitute infectious particles, HFFs were transfected with the obtained BAC DNA using X-tremeGENE transfection reagent (Roche, Mannheim, Germany). Cells were incubated until the appearance of distinct cytopathic changes. Cell culture supernatant containing infectious particles was harvested, centrifuged to remove cellular debris and stored at −80 °C until use.

### Fluorescence-Activated Cell Sorting (FACS) analysis

For FACS analysis of TB40E/IE2eYFP or TB40E/IE2eYFP-delUS28 infected cells HUVECs were harvested at indicated time points post-infection using Accutase Solution for 5–10 min at 37 °C. Cells were washed once with PBS, followed by FBS-containing buffer (2% FBS and 2 mM EDTA in PBS). Next, cells were stained with anti-CXCR4-APC or anti-IgG2ab-APC antibodies in FBS-containing buffer for 1 h at 4 °C. Finally, cells were washed with FBS-containing buffer and fixed with 2% PFA. Samples were analyzed with the BD LSR II Flow Cytometer (BD Biosciences, Franklin Lakes, NJ, USA) and the results were evaluated with FCS Express V3 (De Novo Software, Los Angeles, CA, USA).

### Plasmids

The cDNA encoding hCXCR4 was purchased from the UMR cDNA Resource Center (University of Missouri-Rolla, USA). The cDNA encoding US28wt and US27wt receptor from TB40E strain of HCMV were used. BiLC: Rluc8 plasmids: Rluc8 cDNA was provided by Jonathan A. Javitch, Columbia University, USA. The used plasmids were designed in accordance to the previously described D_2s_R constructs [32]. The cDNAs encoding full-length Rluc8 or fragments for the Rluc8N (residues 1–229) or Rluc8C (residues 230–311) were fused to the C-terminus of the respective receptors by a 24 aa linker in pcDNA5/FRT (Invitrogen). *Emerald* luciferase (Eluc) and *firefly* luciferase (Fluc) split plasmids: The used plasmids were designed in accordance to the described plasmids [37]. Fragments of Eluc, ElucN (residues 1–415) or ElucC (residues 394–542), were C-terminally linked to the respective receptors by a 20 aa linker sequence (4 × SGGGG). Fragments of Fluc, FlucN (residues 1–416) and FlucC (residues 416–550), were C-terminally linked to the respective receptors by a 4 aa linker (SGGG). PCR products were subcloned into pcDNA3.1(+) or pcDNA4/V5-His(B). BRET sensors: CXCR4 was C-terminally fused to the YFP derivative mVenus. G α _i1_-91mVenus was a gift from Jonathan A. Javitch, Columbia University, USA. The Gβ_1_ and Gγ_2_ subunits as well as RlucII-β-arrestin 2 were kindly provided by Michel Bouvier, University of Montreal, Canada. The CAMYEL biosensor was purchased from ATCC, USA. ELISA: CXCR4 cDNA was tagged by N-terminally inserting a FLAG-tag (DYKDDDAAAD) immediately before the start codon and cloned in pcDNA3.1. The truncated version of US28wt, US28Δ300, was constructed by inserting a STOP-codon after residue Gln-300. The DRY-lock mutant of US28, US28DQY, was constructed by mutating the Arg in position 129 of the DRY-motif to Gln as previously described [30, 31]. The double mutant US28Δ300/DQY was constructed by inserting a STOP-codon after residue Gln-300 of the US28DQY mutant. US28wt, US28Δ300, US28DQY, US28Δ300/DQY and NTS1 were N-terminally fused to an HA-tag (YPYDVPDYA) in pcDNA3.1(+). The identity of all plasmids was confirmed by sequencing (LGC Genomics).

### Reagents, antibodies and radioligands

CXCL12 was purchased from PeproTech. Anti-HA, anti-FLAG antibody and secondary peroxidase-conjugated anti-IgG antibody for ELISA as well as Forskolin and Accutase Solution were purchased from Sigma-Aldrich. The anti-human CD184(CXCR4)-APC (clone 12G5) as well as the isotype control anti-mouse IgG2ab-APC were purchased from Miltenyi Biotec. Coelenterazin-h as well as BrightGlo substrate were purchased from Promega. Cell culture reagents for HEK293T, HFF and COS-7 cells were purchased from Gibco/Thermo Fisher Scientific. Medium growth factors for culturing HUVEC was purchased from Lonza. The radiolabelled tracer of CX_3_CL1 was made by applying the oxidative iodination technique to CX_3_CL1, which incorporates ^125^I at the meta-position of tyrosine residue side chains, and the tracer was characterized and purified by RP-HPLC [72]. The 12G5 tracer was instead produced by Bolton-Hunter labelling, which incorporates ^125^I at the amino terminus of the protein.

### BiLC to assess receptor dimerization

HEK293T cells were transiently transfected in 96-well plates with a pair of receptors fused to the C-terminal (Cluc) and N-terminal (Nluc) split of Rluc8, Fluc or Eluc, respectively using TransIT-293 transfection reagent, while the DNA ratio of receptor-Cluc:receptor-Nluc was 1:1. Luminescence was measured 24 h after transfection using the microplate reader Clariostar (BMG Labtech, no emission filter, 2 s recording), following the addition of 100 μl BrightGlo Substrate (Promega) and 5 min incubation at RT.

### BiLC to assess β-arrestin 2 recruitment

HEK293T cells were transiently transfected with ElucN-β-arrestin 2, CXCR4-ElucC and US28, US28 mutants or empty vector (mock) using PEI, while the DNA ratio was 2:1:1. At 48 hours post transfection (hpt), culture medium was replaced by HBSS supplemented with 0.1% BSA. After 30 min incubation at 37 °C/5% CO_2_, cells were stimulated with 100 nM CXCL12 or vehicle (HBSS-0.1%BSA). At 10 min post ligand addition, luminescence was measured using the microplate reader Clariostar (BMG Labtech, no emission filter, 2 s recording), following the addition of 100 μl BrightGlo Substrate and 5 min incubation.

### Bioluminescence resonance energy transfer measurements

In this study, BRET_480-YFP_ also termed BRET^1^ was used for all the following described BRET-based assays. For BRET^1^, one of the proteins is fused to Rluc or brighter forms of Rluc (RlucII/Rluc8) and the other protein is fused to mVenus. Rluc and mVenus serve as energy donors and acceptors, respectively. We used Coelenterazin-h (Promega) as a substrate for the luciferase, which generates light with a maximal emission peak at 480 nm. The emission spectrum of Rluc overlaps with the excitation spectrum of mVenus, which leads to energy transfer and excitation of mVenus, if the two proteins are about less than 10 nm apart from each other. For use of Rluc8 and mVenus a Förster distance (*R*_0_) of 5.55 nm is described [73]. *R*_0_ describes the intermolecular separation of donor and acceptor which allows 50% of the maximal energy transfer. BRET values were collected 5 min after addition of Coelenterazin-h at a final concentration of 5 μM with the microplate reader ClarioStar (BMG Labtech) equipped with the BRET_480-YFP_ filter set (475 ± 30 nm and 535 ± 30 nm). BRET ratio was determined as the ratio of the emitted light by acceptor (filter: filter: 535 ± 30 nm) over donor (475 ± 30 nm).

### BRET titration curves to assess receptor dimerization

For BRET titration experiments a constant amount of the receptor-Rluc8 plasmid (energy donor) was cotransfected with increasing amounts of the receptor-mVenus plasmid (energy acceptor) using PEI. At 2 d post transfection, culture medium was replaced by HBSS complemented with 0.1% BSA and cells were incubated for 30 min at 37 °C/5% CO_2_ before measurement of BRET. To determine the specific BRET signal (net BRET), the BRET signal detected in cells expressing the energy donor only was subtracted from the BRET signal obtained from cells expressing the acceptor and donor. The net BRET values were plotted as a function of the expression level of the acceptor over the expression of the donor for each individual transfection. The expression level of the acceptor was determined by measuring mVenus fluorescence (ex: 497 ± 15 nm, em: 535 ± 30 nm) and the expression level of the donor was determined as emitted light by the donor (filter 475 ± 30 nm).

### BRET-based measurements of G_i_ protein activation

HEK293T cells were cotransfected with a beforehand optimized DNA ratio of CXCR4-Rluc8, Gα_i1_-mVenus, Gβ_1_, Gγ_2_ and US28wt, US28 mutants or empty vector (mock), whereas the DNA-ratio of CXCR4-Rluc8 to US28, US28 mutant or empty vector was 1:1. Cells were transfected using PEI and seeded in 96-well plates at a density of 25,000 cells per well and incubated for 48 h. For the assay, culture medium was replaced by HBSS complemented with 0.1% BSA and cells were incubated for 30 min at 37 °C/5% CO_2_. Cells were treated with 100nM CXCL12 or vehicle (HBSS-0.1% BSA) and BRET was measured 2 min later. To determine the ligand-promoted BRET signal (ΔBRET), BRET signal detected in vehicle-treated cells was subtracted from BRET signal detected in stimulated cells for each transfection.

### BRET-based measurements of β-arrestin 2 recruitment

HEK293T cells were cotransfected with RlucII-β-arrestin 2, CXCR4-Rluc8 and US28, US28 mutants or empty vector (mock) whereas the CXCR4:US28 DNA ratio was 1:1. Cells were transfected using PEI and seeded in 96-well plates at a density of 25,000 cells per well and incubated for 48 h. For the assay, culture medium was replaced by HBSS complemented with 0.1% BSA and cells were incubated for 30 min at 37 °C/5% CO_2_. BRET was measured 5 min after stimulation with endogenous ligands. To determine the ligand-promoted BRET signal (ΔBRET), BRET signal detected in vehicle-treated cells was subtracted from BRET signal detected in stimulated cells for each transfection.

### BRET-based cAMP assay (CAMYEL-sensor)

HEK293T cells were cotransfected with chemokine receptor cDNA and US28wt, US28Δ300 or US28DQY and CAMYEL biosensor at a DNA ratio of 1:1:2 using PEI and seeded into half-area 96-well plates at a density of 1,5 × 10^4^ cells per well. At 48 h after transfection, the culture medium was removed and replaced by HBSS complemented with 0.1% BSA and incubated for 30 min at 37 °C/5%CO_2_. BRET values were collected 15 min after simultaneous treatment with indicated concentrations of CXCL12 and a final concentration of 10 μM Forskolin.

### Confocal laser scanning microscopy

The day before transfection, HEK293T cells were seeded in 6-well plates at a density of 2 × 10^5^ cells/well. Cells were transfected with C-terminally eGFP-tagged CXCR4 and C-terminally mCherry-tagged US28 or empty vector (mock) using TransIT293(MIRUS). Cells were transferred to Poly-L-Lysine coated glass coverslips 24 h after transfection. At 2 days after transfection, cells were washed with PBS and fixed with 4% paraformaldehyde (RotiHistofix,Carl Roth) for 10 min. After washing three times with PBS, the glass coverslips were mounted on microscope slides using Dako Fluorescent Mounting Medium and investigated using a Leica SP5II confocal microscope (Software LAS AF v2.7.3.9723) equipped with Leica hybrid detectors. Excitation energy and gain were set to the same level to make all data set-ups comparable in intensity. Microscopy/Image analysis was performed with support from the Optical Imaging Center Erlangen (OICE). Post image processing (adjusting brightness and contrast) was performed for a better visualization.

### Enzyme linked immunosorbent assay (ELISA)

HEK293T cells were transiently cotransfected with Flag-tagged chemokine receptors and HA-tagged US28 wildtype or mutants or empty vector (mock) at a DNA ratio of 1:1. 24 h after transfection, cells were seeded in Poly-D-Lysine-coated 48-well plates. At 48 hours post transfection the cells were fixed with 4% PFA for 10 min at RT. Cells were permeabilized or not for 5 min in PBS/0.1% TritonX-100 at RT. In separate wells, cells were stained with monoclonal anti-Flag or anti-HA antibody produced in mouse followed by an anti-mouse, IgG-peroxidase conjugated antibody. Absorbance at 492 nm was measured 10 min after incubation in substrate buffer containing 6 mM o-phenylenediamine using the microplate reader ClarioStar (BMG LabTech).

### Data and statistical analysis

All graphs were generated and analyzed using PRISM 6.0 (GraphPad Software, San Diego, CA). Curves were fitted using least square nonlinear regressions assuming a one site hyperbola where K_d_ corresponds to BRET_50_ and B_max_ corresponds to BRET_max_ or linear regression (BRET saturation experiments) or sigmoidal fit (dose-response curves), in which the logIC_50_ and Hill coefficient were free parameters. Statistical analysis was performed using one-way ANOVA with Dunnett’s post hoc test if more than two values were compared with the control or Student’s *t* test if two values were compared.

### Radioligand competition binding assay

Two days before the assays, the calcium phosphate precipitation method was used to transiently transfect cells with pcDNA3.1(+) vectors expressing either CXCR4 or US28, and on the next day, the transfected cells were seeded to 96-well plates. For the competition binding assays, the cells were washed in HEPES buffer (50 mM) supplemented with BSA (5 g/l) and chilled at 5 °C. Unlabeled ligands were added to the cells 5 min before adding the tracer, which was administered at levels leading to 10% tracer binding. Following an incubation period of 3 h at 4 °C, the cells were washed in HEPES buffer with BSA (5 g/l) and NaCl (29.22 g/l) to remove any unbound tracer, and gamma radiation of the remaining tracer was measured.

## Additional files

Additional file 1: Figure S1. Influence of the constitutive activity of US28 on Gα_i/o_ protein-dependent signaling of CXCR4 (a) Basal and CXCL12-induced changes in cAMP levels in CXCR4/US28 coexpressing, CXCR4-only, US28-only or CAMYEL sensor-only expressing HEK293T cells. Data were normalized on signal from mock-cotransfected cells stimulated with 100 nM CXCL12 (0%) or vehicle (100%). Curves represent means ± SEM from three independent experiments, each performed in triplicate wells. (b) For each transfection, 3 × 10^5^ HEK293T cells were transfected with 1 μg cDNA, while amounts of cDNA for CXCR4-Rluc8 US28wt was fixed and the DNA amount for Gα _i1_-mVenus was gradually increased. After stimulation with vehicle or 100 nM CXCL12, BRET was measured. ΔBRET was calculated by subtracting BRET ratios of vehicle-treated cells from BRET ratios of cells treated with ligand for each individual transfection. Comment on Additional file 2: Figure S2b: Data in Fig. 1b was generated using a ratio of 1:1:20 (15 ng:15 ng:300 ng) of CXCR4-Rluc8:US28wt:Gα_i1_-mVenus. We titrated the amount of cDNA for Gα_i1_-mVenus and observed that at a ratio of 1:1:10 the recruitment of Gα_i1_ to CXCR4 is saturated and does not increase with further increasing concentrations of Gα_i1_-mVenus. (TIF 152527 kb)
Additional file 2: Figure S2. US27 does not alter surface expression of CXCR4 in HEK293T cells. HEK293T cells were cotransfected with CXCR4, N-terminally tagged with FLAG, and mock or US27wt (a) Surface expression of CXCR4 was calculated as the signal ratio between permeabilized and non-permeabilized cells (reflected by FLAG-immunoreactivity) and normalized on the surface expression in CXCR4-only expressing cells. (b) The total expression of CXCR was calculated as a factor of FLAG-immunoreactivity in mock-transfected cells. (TIF 138263 kb)
Additional file 3: Figure S3. β-arrestin 2 pool titrations. HEK293T cells were cotransfected with fixed amounts of CXCR4-ElucC and mock (a) or US28wt (b) cDNA and the DNA amount for ElucN-β-arrestin 2 was increased. After stimulation with vehicle or 100 nM CXCL12, luminescence was measured. ΔLuminesence was calculated by subtracting luminescence detected in vehicle stimulated cells from luminescence detected in cells stimulated with ligand for each transfection. Columns represent means ± SEM of at least two independent experiments each performed in quadruplicates. (TIF 130557 kb)
Additional file 4: Figure S4. Assessment of receptor homo- and heterodimerization using *firefly* luciferase (Fluc) and *emerald* luciferase (Eluc) splits. Receptors carrying splits of Fluc (FlucN/FlucC) (a) or Eluc (ElucC/ElucN) (b) at their C-terminus were used. Columns represent mean ± SEM from at least four independent experiments (*n* = 4–6), each performed in quintuplicates. Measured luminescence is represented as a factor of signal from mock-transfected cells. (TIF 131059 kb)

## Abbreviations

AC: Adenylate cyclase
BAC: Bacterial artificial chromosome
BiLC: Bioluminescence complementation
BRET: Bioluminescence resonance energy transfer
EBV: Epstein Barr virus
EC: Endothelial cells
ELISA: Enzyme-linked immunosorbent assay
Eluc: *emerald* luciferase
FACS: Fluorescence-activated cell sorting
FBS: Fetal bovine serum
Fluc: *firefly* luciferase
FRAP: Fluorescence recovery after photobleaching
FRET: Fluorescence resonance energy transfer
HCMV: Human cytomegalovirus
HIV: Human immunodeficiency virus
HSPCs: Hematopoietic stem and progenitor cells
hpi: Hours postinfection
HUVEC: Human umbilical vein endothelial cells
IC_50_: half maximal inhibitory concentration
KSHV: Kaposi's sarcoma-associated herpesvirus
MCP-1: Monocyte chemotactic protein-1
MOI: multiplicity of infection
NTS1: Neurotensin receptor type 1
RANTES: Regulated on activation, normal T-cell expressed and secreted
Rluc8: *Renilla reniformis* luciferase 8
SDF1α: Stromal cell-derived factor-1α
vGPCRs: viral G protein-coupled receptors
